# Combination of a six microRNA expression profile with four clinicopathological factors for response prediction of systemic treatment in patients with advanced colorectal cancer

**DOI:** 10.1371/journal.pone.0201809

**Published:** 2018-08-03

**Authors:** Maarten Neerincx, Dennis Poel, Daoud L. S. Sie, Nicole C. T. van Grieken, Ram C. Shankaraiah, Floor S. W. van der Wolf - de Lijster, Jan-Hein T. M. van Waesberghe, Jan-Dirk Burggraaf, Paul P. Eijk, Cornelis Verhoef, Bauke Ylstra, Gerrit A. Meijer, Mark A. van de Wiel, Tineke E. Buffart, Henk M. W. Verheul

**Affiliations:** 1 Department of Medical Oncology, VUmc Cancer Center Amsterdam, VU University Medical Center, Amsterdam, The Netherlands; 2 Department of Pathology, VUmc Cancer Center Amsterdam, VU University Medical Center, Amsterdam, The Netherlands; 3 Department of Radiology, VUmc Cancer Center Amsterdam, VU University Medical Center, Amsterdam, The Netherlands; 4 Department of Pathology, Spaarne Gasthuis, Hoofddorp, The Netherlands; 5 Department of Surgical Oncology, Erasmus Medical Center Cancer Institute, Rotterdam, The Netherlands; 6 Department of Pathology, The Netherlands Cancer Institute, Amsterdam, The Netherlands; 7 Department of Epidemiology and Biostatistics, VUmc Cancer Center Amsterdam, VU University Medical Center, Amsterdam, The Netherlands; 8 Department of Mathematics, VU University, Amsterdam, The Netherlands; Universitat de Barcelona, SPAIN

## Abstract

**Background:**

First line chemotherapy is effective in 75 to 80% of patients with metastatic colorectal cancer (mCRC). We studied whether microRNA (miR) expression profiles can predict treatment outcome for first line fluoropyrimidine containing systemic therapy in patients with mCRC.

**Methods:**

MiR expression levels were determined by next generation sequencing from snap frozen tumor samples of 88 patients with mCRC. Predictive miRs were selected with penalized logistic regression and posterior forward selection. The prediction co-efficients of the miRs were re-estimated and validated by real-time quantitative PCR in an independent cohort of 81 patients with mCRC.

**Results:**

Expression levels of miR-17-5p, miR-20a-5p, miR-30a-5p, miR-92a-3p, miR-92b-3p and miR-98-5p in combination with age, tumor differentiation, adjuvant therapy and type of systemic treatment, were predictive for clinical benefit in the training cohort with an AUC of 0.78. In the validation cohort the addition of the six miR signature to the four clinicopathological factors demonstrated a significant increased AUC for predicting treatment response *versus* those with stable disease (SD) from 0.79 to 0.90. The increase for predicting treatment response *versus* progressive disease (PD) and for patients with SD *versus* those with PD was not significant. in the validation cohort. MiR-17-5p, miR-20a-5p and miR-92a-3p were significantly upregulated in patients with treatment response in both the training and validation cohorts.

**Conclusion:**

A six miR expression signature was identified that predicted treatment response to fluoropyrimidine containing first line systemic treatment in patients with mCRC when combined with four clinicopathological factors. Independent validation demonstrated added predictive value of this miR-signature for predicting treatment response *versus* SD. However, added predicted value for separating patients with PD could not be validated. The clinical relevance of the identified miRs for predicting treatment response has to be further explored.

## Introduction

Colorectal cancer (CRC) is the third most common cancer worldwide and has a 5 year survival of only 13% when disseminated [1, 2]. Approximately 20% of patients present with metastatic disease (mCRC) and another 25–30% will develop metastases after initial surgical resection of their primary tumor [3]. For patients with resectable metastases several localized treatment options are available [4]. Patients with initially irresectable metastases are treated with systemic therapy consisting of a fluoropyrimidine (5-fluorouracil or capecitabine), oxaliplatin and/or irinotecan and a biological agent (bevacizumab, cetuximab or panitumumab) in a neoadjuvant or palliative setting. First line systemic therapy induces a treatment response or disease stabilization in 75–80% of patients with mCRC [5–8]. Consequently, 20–25% of patients receive systemic treatment without any benefit while causing multiple toxicities.

Predictive biomarkers for treatment benefit prior to the start of treatment can prevent the use of ineffective treatment regimens, avoid unnecessary toxicity and minimize the delay of treatment with alternative effective regimens. At this moment RAS mutation status is the only routine biomarker to predict treatment benefit in patients with mCRC [9, 10]. Small non-coding microRNAs (miRs) are an attractive source for predictive biomarker development as they post-transcriptionally regulate many target genes involved in carcinogenesis. MiRs are deregulated in the tumor genome. They are frequently located at genomic regions with gains or losses in the tumor genome and abnormalities in miR processing genes or proteins can enhance cancer development [11, 12]. As miRs are relatively resistant to degradation in formalin fixed and paraffin embedded (FFPE) material as well as in blood, they are suited for the use as biomarkers in clinical practice [13–15]. Indeed, miR expression levels distinguishes different tumor types from normal tissue and have been identified as potential biomarkers for mCRC [16, 17]. Currently, miRs with prognostic and predictive value have been identified for localized and metastasized CRC [18–25]. However, these miRs were identified by probe based methodologies and consequently these studies were inherently restricted to a limited number of miRs. We previously identified 222 tumor specific miRs differentially expressed between CRC tumor tissue and corresponding normal tissue by an unbiased whole genome approach using next generation sequencing (NGS) [17]. Here, we used NGS to identify a predictive miR expression profile based on these tumor specific miRs for patients with mCRC treated with first line fluoropyrimidine-based treatment regimens and examined its performance in an independent patient group.

## Materials and methods

### Patients and tumor samples

A total of 169 patients with mCRC were included. Patients with known Lynch syndrome or CRC secondary to inflammatory bowel disease or patients who were treated with neoadjuvant radiotherapy or with chemotherapy within 6 months before tumor resection were excluded for this study. Samples were collected from consecutive patients who entered the VU University Medical Center from July 2003 until November 2011 or the Spaarne Hospital from January 2005 until December 2010. Retrospective collection, storage and use of patient data were approved by the Medical Ethical Committee of the VU University Medical Center. Written or verbal informed consent was not obtained due to the retrospective nature of the study in concordance with Dutch law. All patients were deceased or lost to follow-up. Collection, storage and use of tumor samples were performed in accordance with the Code for proper secondary use of human tissue in The Netherlands [26].

Samples of primary as well as metastatic tumor tissue were included as miR profiles from metastatic tumor tissue only differ by 0,5% from their corresponding primary tumor [17]. Patients were synchronously metastasized in 62.7% (stage IV at presentation) and metachronously metastasized in 36.7% (stage I-III at presentation) of the cases (Table 1). Patients were treated with first line systemic treatment for mCRC for at least 6 weeks. Treatment consisted of a fluoropyrimidine (infusional 5-fluorouracil or oral capecitabine), oxaliplatin, irinotecan or combinations. Additional anti-VEGF (bevacizumab) or anti-EGFR monoclonal antibodies (cetuximab or panitumumab) were allowed. Computed tomography or ultrasound imaging was performed before and during treatment to evaluate response rates in all 169 patients. Samples were divided into a training and validation cohort based on the availability of fresh-frozen tumor samples.

**Table 1:** Baseline characteristics of the 169 patients with advanced colorectal cancer included in the training and validation cohorts.

	Training cohort (N = 88)	Validation cohort (N = 81)	P value^1^
Sex—N (%)			1.00
Female	32 (36.4)	30 (37.0)	
Male	56 (63.6)	51 (63.0)	
Age—yr			0.01
Median (range)	65 (41–88)	61 (37–81)	
Primary tumor location—N (%)			0.005
Rectal	9 (10.2)	20 (24.7)	
Left sided	53 (60.2)	30 (37.0)	
Right sided	26 (29.5)	31 (38.3)	
TNM-stage at time of diagnosis^2^ - N (%)			0.21
Stage I	3 (3.4)	1 (1.2)	
Stage II	10 (11.4)	13 (16.0)	
Stage III	23 (26.1)	12 (14.8)	
Stage IV	52 (59.1)	54 (66.7)	
Missing data	0	1 (1.2)	
Primary tumor differentiation^3^ - N (%)			0.99
Well	1 (1.1)	1 (1.2)	
Moderate	68 (77.3)	53 (65.4)	
Poor	19 (21.6)	15 (18.5)	
Missing data	0	12 (14.8)	
Prior adjuvant therapy for localized CRC—N (%)			0.001
No	68 (77.3)	77 (95.1)	
Yes	20 (22.7)	4 (4.9)	
Prior adjuvant therapy for advanced CRC^4^ - N (%)			1.00
No	85 (96.6)	78 (96.3)	
Yes	3 (3.4)	3 (3.7)	
Liver metastases only—N (%)			0.04
No	48 (54.5)	57 (70.4)	
Yes	40 (45.5)	24 (29.6)	
LDH—N (%)			0.86
Normal (<250 ng/ul)	20 (22.7)	23 (28.4)	
Elevated (≥250 ng/ul)	52 (59.1)	55 (67.9)	
Missing data	16 (18.2)	3 (3.7)	
CEA—N (%)			1.00
Normal (<5 ng/ul)	16 (18.2)	17 (21.0)	
Elevated (≥5ng/ul)	61 (69.3)	61 (75.3)	
Missing data	11 (12.5)	3 (3.7)	

^1^ P values were calculated with Fisher’s exact test, except for age which was calculated with the unpaired t-test, and primary tumor location, TNM stage and primary tumor differentiation which were calculated with the chi-square test

^2^ Stage IV was defined as metastatic disease diagnosed within 30 days of resection of the primary tumor.

^3^ Signet cell differentiation was classified as poorly differentiated

^4^ Macroscopic disease free after local treatment for metastatic disease, preceding first line treatment

Abbreviations: LDH, lactate dehydrogenase, CEA, carcinoembryonic antigen

Fresh frozen tumor samples were available for 103 patients. In 15 samples tumor cell content was less than 70% and therefore these were excluded prior to the analysis to enhance the selection of tumor specific miRs during classifier development. The 88 training samples included 80 primary tumors, 5 metastases and 3 local recurrences and were directly frozen after surgery.

FFPE tumor samples were available for 88 patients. Seven samples were not evaluable due to low RNA quantity or inability to amplify the RNA with RT-qPCR and were excluded prior to the analysis. The 81 validation samples included 54 primary tumor resection specimens, 26 primary tumor biopsies obtained before start of systemic treatment and 1 metastasis. No minimal tumor cell percentage was required for inclusion in the validation cohort, but all contained >40% tumor cells.

### Clinical and pathological factors

Clinical and pathological data with known predictive or prognostic value were collected. Data on the use of local treatment modalities for metastatic disease after start of systemic treatment and the total number of different systemic treatment regimens were collected as well. Potential predictive factors for tumor response included; age at start of systemic treatment for advanced disease (continuous variable), primary tumor differentiation (well or moderate *versus* poor or with signet cell differentiation), previous adjuvant treatment (either for localized CRC or after local treatment for metastases) (yes *versus* no) and treatment regimen (fluoropyrimidine mono-therapy *versus* oxaliplatin containing regimens *versus* irinotecan containing regimens). Primary tumor differentiation grade was missing for 12 tumor samples in the validation cohort because the primary tumor was not resected and the pretreatment biopsies did not yield enough material to reliably determine the differentiation grade.

Additional potential prognostic factors for progression free survival (PFS) included initial tumor stage (synchronous *versus* metachronous disease, with synchronous and metachronous disease defined as distant metastases occurring respectively within and beyond 30 days of primary diagnosis of CRC), metastatic tumor load (liver metastases only *versus* involvement of other organs), lactate dehydrogenase (LDH) (normal *versus* elevated), carcinoembrionic antigen (CEA) (normal *versus* elevated) and the intention of the applied treatment (palliative *versus* neoadjuvant). An overview of the clinicopathological data is given in Table 1.

Local treatment modalities included resection of metastases, radiofrequent ablation, stereotactic radiotherapy, trans-arterial chemoembolization and radio-embolisation procedures. Discontinuation of a drug in case of combination therapy was not considered as start of a different treatment regimen. Restart of a treatment regimen without interim objective progressive disease (PD) was considered as treatment continuation. Restart of a treatment regimen after a treatment free interval with interim objective PD was considered as a new regimen. An overview of the treatment schedules of the patients included in the training and validation cohorts is given in Table 2.

**Table 2:** Treatment characteristics and response evaluation of the patients in the training and validation cohorts.

	Training cohort (N = 88)	Validation cohort (N = 81)	P value^1^
First line treatment—N (%)			0.65
Neoadjuvant	12 (13.6)	9 (11.1)	
Palliative	76 (86.4)	72 (88.9)	
First line treatment scheme—N (%)			0.23
5-FU monotherapy	23 (26.1)	14 (17.3)	
Oxaliplatin-based regimens	51 (58.0)	57 (70.4)	
Irinotecan-based regimens	14 (15.9)	10 (12.3)	
Use of first line Bevacizumab—N (%)			0.35
No	49 (55.7)	51 (63.0)	
Yes	39 (44.3)	30 (37.0)	
Use of first line Cetuximab or Panitumumab -N (%)			0.72
No	83 (94.3)	78 (96.3)	
Yes	5 (5.7)	3 (3.7)	
Number of systemic treatment regimens—N (%)			0.74
1	88 (100.0)	81 (100.0)	
2	54 (61.4)	55 (67.9)	
3	30 (34.1)	33 (40.7)	
≥4	10 (11.4)	13 (16.0)	
Local treatment for advanced disease after baseline—N (%)			0.68
No	72 (81.8)	69 (85.2)	
Yes	16 (18.2)	12 (14.8)	
Best response to first line treatment—N (%)			0.45
Complete response (CR)	0	2 (2.5)	
Partial response (PR)	43 (48.9)	36 (44.4)	
Stable disease (SD)	27 (30.7)	28 (34.6)	
Progressive disease (PD)	18 (20.5)	15 (18.5)	
PFS of first line treatment–months (median, range)			0.46
Overall	7.8 (1.3–79.6)	7.4 (1.0–25.1)	
CR + PR	10.2 (4.1–79.6)	9.1 (3.5–25.1)	
SD	6.6 (2.9–25.6)	7.1(2.4–21.7)	
PD	2.0 (1.3–3.0)	2.1 (1.0–5.4)	
Survival—months (median, range)			0.16
Overall	21.0 (1.7–79.6)	16.4 (3.5–114.3)	
CR + PR	27.4 (8.5–79.6)	21.0 (3.5–75.5)	
SD	16.9 (2.9–58.2)	15.1 (3.7–114.3)	
PD	7.2 (1.7–41.0)	6.6 (4.1–36.6)	

^1^ P values were calculated with Fisher’s exact test, except for treatment scheme and best response which were calculated with the chi-square test and survival which was calculated with the log rank test

Abbreviations: PFS, progression free survival

### Outcome parameters

Treatment response was evaluated by two radiologists (FSWvdW and JHTMvW) and categorized as complete response (CR), partial response (PR), stable disease (SD) and progressive disease (PD) according to the Response Evaluation Criteria In Solid Tumors (RECIST version 1.1) [27]. When imaging results were difficult to interpret, independent re-evaluation was performed. PFS was defined as time between start of first line treatment until disease progression on imaging. When documentation of progression on follow up imaging was not available, a rise in CEA level was used instead to evaluate PFS. If progression was not observed during treatment, the date of last imaging was used as follow-up date for the survival analyses. Overall survival (OS) was defined as time between start of first line treatment until death from any cause. Survival dates were collected from the local authorities (Gemeentelijke Basis Administratie, GBA). Follow up ended on March 1^st^ 2015. An overview of outcome parameters of the patients included in the training and validation cohorts is given in Table 2.

### RNA isolation

Of all 169 tumor tissues 4 μm sections were made, stained with hematoxylin and eosin (H&E) and evaluated by a GI pathologist (NCTvG or GAM) for tumor cell content. Of the 88 fresh frozen tumor tissues, areas with the highest tumor cell density were selected and the remaining tissue was macrodissected and removed from the tissue specimen as previously described [17]. Total RNA was isolated using TRIzol (Invitrogen, Carlsbad, CA, USA) following the manufacturer’s guidelines with some modifications [17]. Of the 81 FFPE samples, areas with the highest tumor cell density were macrodissected from 20-μm sections. RNA was isolated using the RecoverAll Total Nucleic Acid Isolation Kit (Applied Biosystems, Foster City, CA, USA) according to the manufacturer’s guidelines. RNA quantity of the 169 samples was determined with a Nanodrop 2000 (Thermo Scientific, MA, USA).

### Next generation sequencing and data processing

Next generation sequencing (NGS) using Illumina’s TruSeq Small RNA Sample Preparation protocol and data filtering were performed as previously described [17]. Illumina’s TruSeq Small RNA Sample Preparation protocol was used for the generation of cDNA libraries. These libraries were amplified on the flow cells with Illumina’s cluster station (Illumina Inc, San Diego, CA, USA) and sequenced using Illumina’s HiSeq 2000 (Illumina Inc, San Diego, CA, USA). Obtained sequence reads were first quality trimmed, resulting in a >99.9% probability of a correctly identified base of the remaining nucleotides. Secondly, the reads were clipped for adaptor sequences. Thirdly, reads with identical sequences were compiled and counted, resulting in only unique sequences. Finally, each unique sequence was mapped to the reference genome (browser hg19) and only those alignments of at least 18 nucleotides and a maximum of 2 mismatches were retained.

After data filtering steps, the deep sequencing reads were quantified by mapping them against the known precursor sequences from mirbase v.19 and the novel candidate precursor sequences resulting from our previous work [17]. Reads that map equally well to the positions of multiple mature miRs were added to the read counts of those mature miRs. Read counts of identical mature miRs mapping to related precursors (e.g. hsa-mir-7-1, hsa-mir-7-2, hsa-mir-7-3) were averaged. Genome data has been deposited at the European Genome-phenome Archive (EGA, http://www.ebi.ac.uk/ega/) which is hosted at the European Bioinformatics Institute (EBI), under accession number EGAS00001001127.

### Reverse transcription quantitative PCR

Reverse transcription quantitative (RT-q) PCR for miRs was performed using the miRCURY LNA^™^ Universal RT microRNA PCR system (Exiqon A/S, Vedbaek, Denmark) according to the manufacturer’s instructions. The synthetic spike-in UniSp6 was replaced with nuclease free water (Promega, WI, USA). Complement cDNA was diluted 1:40. RT-qPCR was performed in duplicate according to the manufacturer’s instructions and run on a CFX96 RT-PCR detection system (Bio Rad, CA, USA). For individual miR assays Exiqon LNA primer sets were used (Exiqon A/S, Vedbaek, Denmark). Average Cq values were normalized to miR-16-5p as reference miR [28, 29]. RT-qPCRs were repeated with 15 ng input RNA if the standard deviation of the duplicate was above 0.6 or when no expression was observed. Colorectal adenocarcinoma cell line HT29 was used as positive control for the assays with miR-16-5p, miR-17-5p, miR-20a-5p, miR-92a-3p and miR-98-5p. For miR-30a-5p the head and neck squamous cell carcinoma cell line SCC120 and for miR-92b-3p colorectal adenocarcinoma cell line H630 were used as positive control. A melt curve analysis was performed for amplification specificity of each individual target per sample.

### Statistical analysis

Read counts of the samples of the training set were normalized using edgeRs TMM method [30]. Class prediction and differential expression analyses were performed for miRs expressed in at least 5 samples. Analyses were performed for all identified miRs as well as for the previously identified subgroup of 222 tumor specific miRs [17].

#### Treatment response

Predictive covariates for treatment response included age of the patient, primary tumor differentiation, prior use of adjuvant therapy and the type of systemic treatment regimen. Global test statistics corrected for these covariates were used to test whether miR expression levels were associated with response to treatment [31].

Class prediction and miR selection were performed using the GRridge package (version 1.5) in the statistical programming language R [32]. Weighted logistic ridge regression and posterior forward selection were performed to select the miRs predictive of treatment response [32]. The total read count and standard deviation of each miR were used as co-data to provide unbiased weights for prediction and miR selection [32], which lead to a preference for higher expression levels. Tumor samples of the training set were divided into patients with clinical benefit (CR, PR or SD) *versus* patients with PD. Differential expression analyses between the patients with clinical benefit and those with PD were performed by testing the additive value of a miR with respect to the aforementioned predictive covariates in a logistics regression setting, followed by a Benjamin-Hochberg correction for multiple testing. FDR values of <0.1 were considered significant.

#### Survival

Prior to analysis tumor stage at diagnosis (synchronous disease *versus* metachronous disease), liver metastases only (yes *versus* no) and intention of the applied treatment (palliative *versus* neoadjuvant) were added as prognostic covariates for survival analyses. Data on LDH and CEA levels were missing for 16 and 11 patients respectively and not included as covariates (Table 1). Differential expression analyses for PFS were performed as described above, but with Cox regression instead of logistic regression. FDR values of <0.1 were considered significant.

#### Independent validation

To minimize the influence of selection bias on the effect size of the prediction co-efficients of the selected miRs, the model resulting from the training cohort was re-estimated in the independent sample set. Model co-efficients and fold changes were calculated with multivariate logistic regression analysis using Akaike’s information criterion (AIC)-based backward selection. The training cohort was divided into patients with treatment response (CR or PR), patients with SD and patients with PD [33]. Data on primary tumor differentiation was missing for 12 patients in the validation cohort and missing data was included as separate level of this covariate. Added predictive value of selected miRs to clinicopathological factors was tested using DeLong’s method for comparing the AUCs of paired ROC curves, as implemented in the R-package ‘pROC’ [34, 35], with a p-value < 0.05 regarded as significant added predictive value. Differential expression of the selected predictive miRs was tested by using the Wilcoxon rank sum test, with a p-value < 0.05 regarded as a significant different expression level.

## Results

### Patient and tumor sample characteristics

Of the 88 samples in the training cohort, 81 samples (92%) were chemotherapy naive and 7 samples (8%) were collected after a > 6 months chemotherapy free period. All 81 samples of the validation cohort were chemotherapy naive. None of the patients in the training cohort and 10 patients in the validation cohort received neoadjuvant radiotherapy on their primary rectal tumors, but included tumor biopsies were obtained before start of radiotherapy. Patients in the training cohort were significantly older than patients in validation cohort (median 65 years *versus* 61 years respectively, p = 0.01), had a significantly different tumor distribution throughout the colon with less rectal tumors (10.2% *versus* 24.7% respectively, p = 0.005), more often had liver metastases only (45.5% *versus* 29.6% respectively, p = 0.04) and more often received prior adjuvant chemotherapy (22.7% *versus* 4.9% respectively, p = 0.001). Other patient characteristics were not significantly different between the two groups (Table 1). Patients received fluoropyrimidine-based treatment as first line treatment for mCRC, except for 1 patient in the training cohort which received fluoropyrimidine containing adjuvant treatment for localized disease and was treated with irinotecan monotherapy (Table 2). Tumor cell content of the samples from the validation set ranged between 40% and 80%, with 55/81 (67.9%) of the samples containing 70% or more tumor cells.

### MiR expression profiles obtained by next generation sequencing

The number of nucleotide sequences (reads) obtained by NGS of the 88 fresh-frozen tumor samples ranged from 6.114.932 to 74.313.067 reads per sample, with a median of 9.179.594 reads per sample. After data filtering steps 541.909.004 nucleotide sequences of at least 18 nucleotides mapped to the reference genome with a maximum of two mismatches, which was 61.0% of the initial total number of reads. In these sequences 2567 unique mature miR sequences were identified, consisting of 457 novel candidate miR sequences and 2110 miR sequences known according to miRbase version 19. The read counts of these 2567 miRs ranged from 1 to 80.932.357. Of these miRs, 2113 miRs were expressed in at least 5 of the 88 samples and were included for further analyses. These miRs included 221 of the previously identified 222 tumor specific miRs [17].

### Six-miR expression profile combined with four clinicopathological factors is predictive for clinical benefit on first line chemotherapy

**Fig 1:**
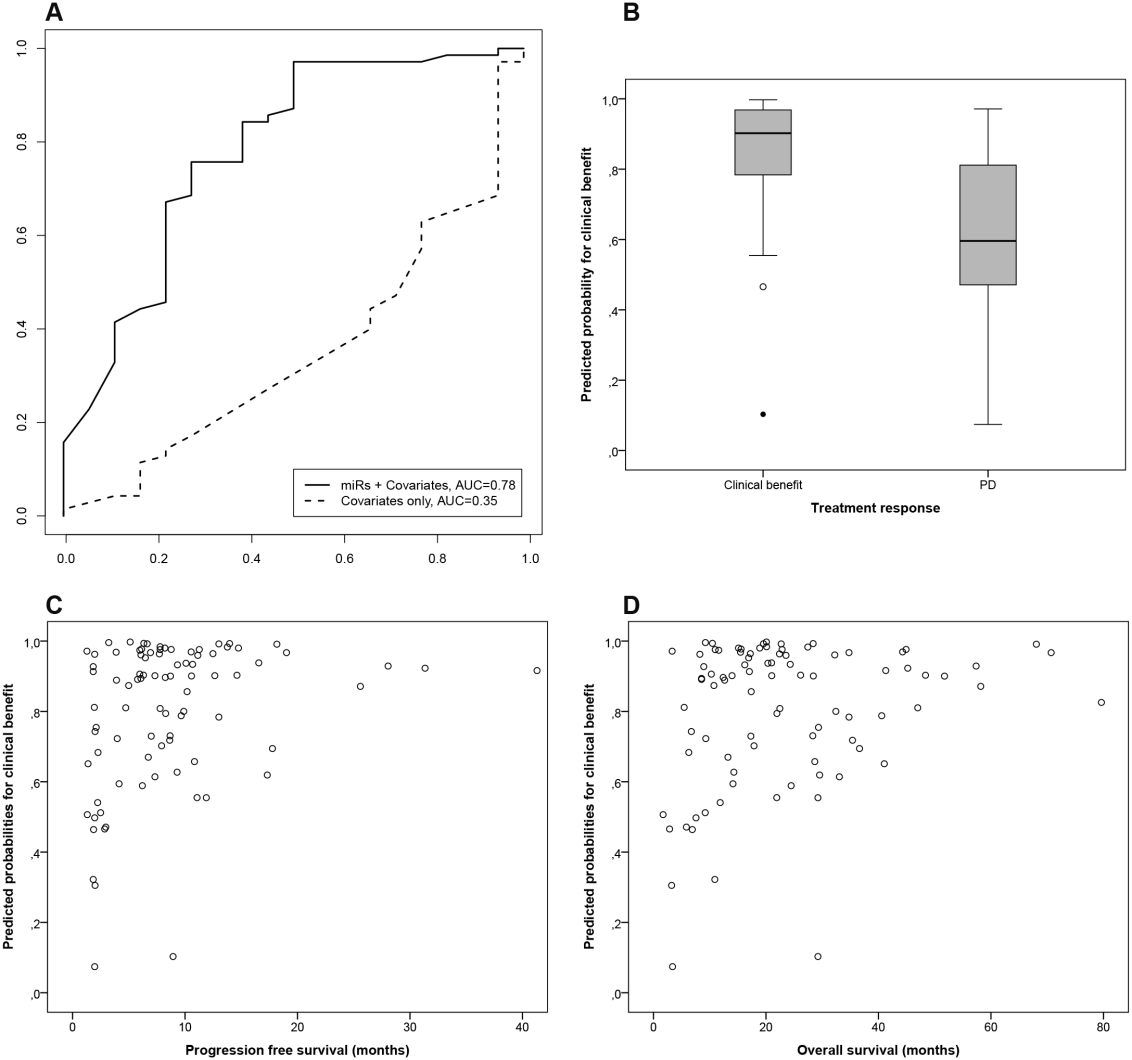
Performance of the classifier in the training cohort. **(A)** Receiver operating characteristic (ROC) curve of six-miR classifier predictive for response to first line systemic treatment for patients with mCRC based on the training cohort (n = 88), resulting in an area under the curve (AUC) of 0.78. Included in the classifier are miR-17-5p, miR-20a-5p, miR-30a-5p, miR-92a-3p, miR-92b-3p and miR-98-5p and four clinicopathological covariates; prior use of adjuvant therapy, the type of systemic treatment regimen, age and primary tumor differentiation. When excluding the miRs from the prediction algorithm the AUC drops to 0.35. The false positive rate (1-specificity) is depicted on the x-axis and, the sensitivity is depicted on the y-axis. **(B)** Boxplot of the internal cross validated predicted probabilities for clinical benefit. The median predicted probability for the 70 patients with clinical benefit was 0.90 (IQR: 0.77–0.97). For the 18 patients with progressive disease the median predicted probability for clinical benefit was 0.60 (IQR: 0.47–0.84). Predicted probabilities were calculated using the expression levels of the six selected miRs and four clinicopathological covariates. **(C)** Correlation between the predicted probabilities for clinical benefit (y-axis) with progression free survival (x-axis) of the training cohort. There is a significant correlation of 0.30 (spearman’s rho) (p = 0.006). **(D)** Correlation between the predicted probabilities for clinical benefit (y-axis) with overall survival (x-axis) of the training cohort. There is a correlation of 0.19 (spearman’s rho), which is not significant (p = 0.08).

After normalization of the read counts, class prediction for clinical benefit compared to PD was performed with all 2113 miRs and with the 221 tumor specific miRs. Age, primary tumor differentiation, prior use of adjuvant therapy and the type of systemic treatment regimen were included as predictive covariates. Using global test statistics, the association between all 2113 miRs with clinical benefit resulted in a non-significant p-value of 0.07. The association of the 221 tumor specific miRs with clinical benefit was much stronger and resulted in a significant correlation between miR expression and response to treatment (p = 0.008). Therefore, expression levels of non-tumor specific miRs did not add predictive value for clinical benefit to the tumor specific miRs. Using penalized logistic regression, six miRs were selected to build the predictive classifier; miR-17-5p, miR-20a-5p, miR-30a-5p, miR-92a-3p, miR-92b-3p and miR-98-5p. Combination of the expression patterns of these six miRs together with the four clinicopathological covariates resulted in a discriminatory performance between patients with and without clinical benefit from first line treatment, with an AUC of 0.78 (Fig 1A). Using the predictive classifier without the selected miRs resulted in a non-predictive AUC of 0.35 (Fig 1A). Probabilities for clinical benefit for individual patients were calculated and cross-validated using individual expression levels of the six miRs and individual values for the four clinicopathological covariates. The median predicted probability for clinical benefit of the 70 patients with clinical benefit was 0.90 (IQR 0.77–0.97) (Fig 1B). For the 18 patients with PD the median predicted probability for clinical benefit was 0.60 (IQR 0.47–0.84) (Fig 1B). Two patients with actual clinical benefit had a low predicted probability for clinical benefit (0.47 and 0.10 respectively). Both patients had SD as best response to first line treatment. The correlation between the predicted probabilities for clinical benefit with PFS and OS are shown in Fig 1C and 1D. The correlation with PFS is moderate (spearman’s rho = 0.30), although significant (p = 0.006). The correlation with OS (spearman’s rho = 0.19) is not significant (p = 0.08). A low predicted probability for clinical benefit has a high negative predictive value for worse prognosis (PFS as well as OS), while a high predicted probability for clinical benefit has a low positive predictive value for a good prognosis (Fig 1C and 1D). To evaluate the individual discriminatory value of the 221 tumor specific miRs, differential expression analyses including the four predictive covariates were performed between patients with clinical benefit and those with PD. Seventeen miRs were significantly different expressed (FDR <0.1) between patients with clinical benefit *versus* patients with PD during first line treatment (Table 3). Of the six selected miRs, miR-17-5p, miR-20a-5p and miR-92a-3p were significantly upregulated in the tumors of the patients with clinical benefit on first line treatment compared to those of the patients with PD. MiR-30a-5p, miR-92b-3p and miR-98-5p were not significantly different expressed between the two groups (Table 3).

**Table 3:** Differential expression analysis of 20 miRs in the training cohort. The multivariate logistic regression analysis is based on the tumor specific miRs (N = 221). For each miR, p-values and FDR values of the multivariate logistic regression analysis and total number of reads are shown. Seventeen miRs were significantly differently expressed between patients with clinical benefit *versus* progressive disease on first line systemic treatment. Three of the 6 miRs included in the prediction model were not significantly differently expressed but are included as well. The 6 miRs of the prediction model are shown in bold.

MiR	p-value	FDR	Read count
hsa-miR-592	0.000	0.024	34304
hsa-miR-92a-1-5p	0.000	0.036	7030
**hsa-miR-20a-5p**	**0.000**	**0.036**	**495181**
**hsa-miR-92a-3p**	**0.001**	**0.072**	**11259402**
hsa-miR-548ar-5p	0.002	0.072	420
**hsa-miR-17-5p**	**0.002**	**0.072**	**347413**
hsa-miR-2467-5p	0.002	0.072	2908
hsa-miR-29c-5p	0.003	0.078	3808
hsa-miR-3200-3p	0.003	0.078	2950
hsa-miR-29b-2-5p	0.004	0.081	752
hsa-miR-548h-5p	0.004	0.086	590
hsa-miR-3912-3p	0.005	0.086	1230
hsa-chr16_35996-5p	0.006	0.094	55
hsa-miR-548aj-5p	0.007	0.094	513
hsa-miR-4745-5p	0.007	0.094	78
hsa-miR-548x-5p	0.007	0.094	507
hsa-miR-548g-5p	0.007	0.094	508
**hsa-miR-92b-3p**	**0.010**	**0.113**	**652188**
**hsa-miR-98-5p**	**0.011**	**0.118**	**713894**
**hsa-miR-30a-5p**	**0.059**	**0.332**	**1010198**

### Prognostic value of the six-miR expression profile

Using global test statistics, the miR expression of all 2113 miRs as well as the 221 tumor specific miRs were significantly associated with PFS. Again the association of the tumor specific miRs was stronger (p = 0.02 *versus* 0.01 respectively). To evaluate the prognostic value of the individual miRs, a multivariate cox-regression analysis for PFS was performed. Included covariates were the four predictive covariates used for treatment response analyses, with the addition of three prognostic covariates; initial tumor stage, liver metastases only and neoadjuvant *versus* palliative first line treatment. None of the six miRs were individually significantly associated with PFS (FDR <0.1), although miR-17-5p, miR-30a-5p and miR-92b-3p showed p-values smaller than 0.05.

Differential expression analyses for OS were not performed as global test associations of the miRs with OS were not significant.

### Performance of the six-miR expression profile and the four clinicopathological factors in the independent validation set

The performance of the predictive classifier including the expression levels of miR-17-5p, miR-20a-5p, miR-30a-5p, miR-92a-3p, miR-92b-3p and miR-98-5p and the four clinicopathological covariates was evaluated in an independent validation cohort of 81 tumor samples. The classifier was re-estimated by dividing the patients of the validation cohort into patients with treatment response (CR or PR), patients with SD and patients with PD. Three comparisons were made; 1) patients with response *versus* patients with PD, 2) patients with SD *versus* patients with PD and 3) patients with response *versus* patients with SD.

**Fig 2:**
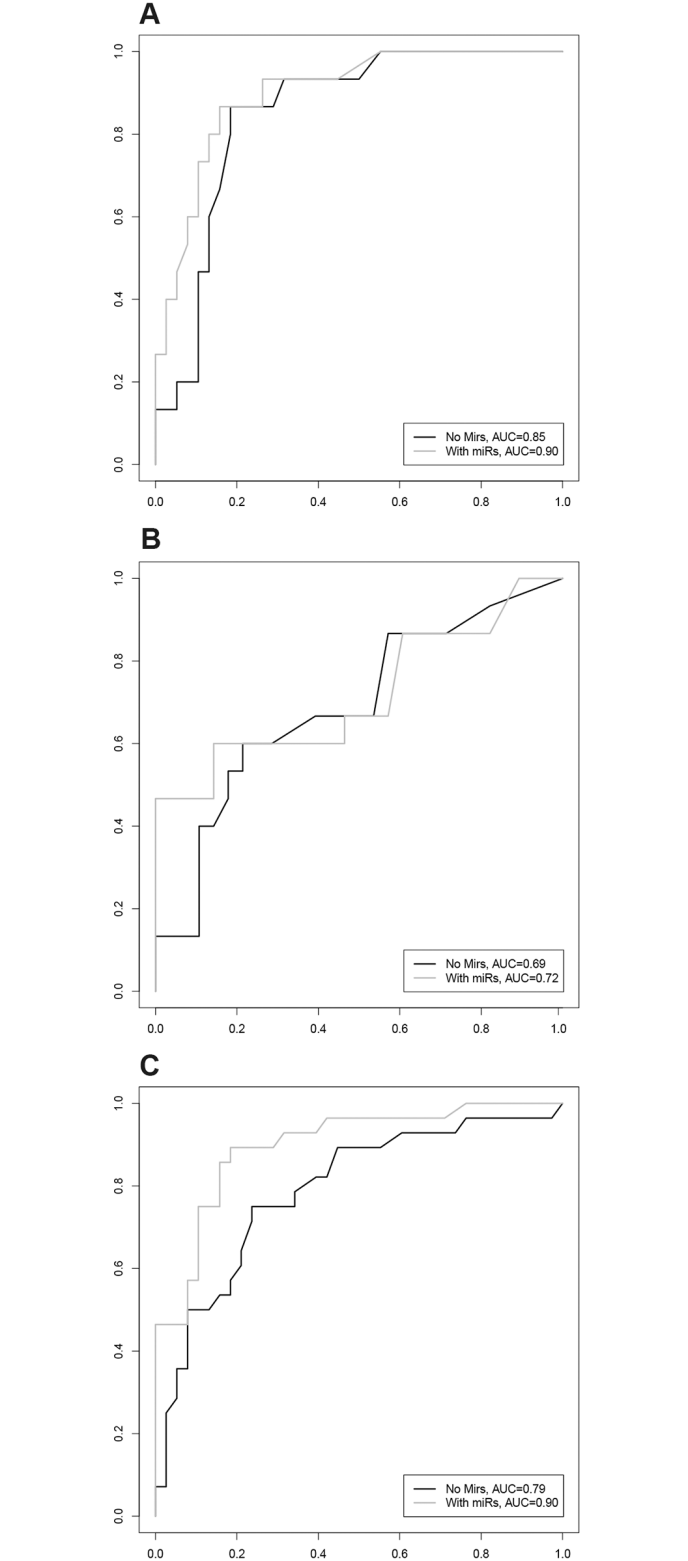
Performance of the classifier in the validation cohort. **(A)** ROC curve of the predictive classifier in the validation cohort for patients with PR or CR on first line systemic treatment (n = 38) compared to patients with PD (n = 15). Included in the classifier are miR-92a-3p, miR-92b-3p and four clinicopathological covariates. On the x-axis the false positive rate (1-specificity) is depicted, on the y-axis the sensitivity is depicted. The AUC of the model for predicting treatment response without miRs is 0.85, compared to 0.90 when including miR-92a-3p and miR-92b-3p to the model, this difference is not significant (p = 0.12). **(B)** ROC curve of the predictive classifier in the validation cohort for patients with SD on first line systemic treatment (n = 28) compared to patients with PD (n = 15). Included in the classifier are miR-30a-5p and therapy regimen. On the x-axis the false positive rate (1-specificity) is depicted, on the y-axis the sensitivity is depicted. The AUC of the model for predicting SD without miRs is 0.69, compared to 0.72 when including miR-30a-5p to the model, this difference is not significant (p = 0.37). **(C)** ROC curve of the predictive classifier in the validation cohort for patients with PR or CR on first line systemic treatment (n = 38) compared to patients with SD (n = 28). Included in the classifier are miR-17-5p, miR-92a-3p, miR-92b-3p and miR-98-5p and differentiation grade of the primary tumor. On the x-axis the false positive rate (1-specificity) is depicted, on the y-axis the sensitivity is depicted. The AUC of the model for predicting treatment response without miRs is 0.79, which increased significantly to 0.90 when including miR-17-5p, miR-92a-3p, miR-92b-3p and miR-98-5p to the model (p = 0.02).

When re-estimating the model on the patients with treatment response *versus* the patients with PD, treatment response was predicted with an AUC of 0.90 with the classifier including miR-92a and miR-92b (Fig 2A). When excluding these miRs from the model, the AUC dropped to 0.85 (Fig 2A), this difference was not significant (p = 0.12) indicating that expression levels of the six selected miRs added no predictive value to clinicopathological factors alone for this comparison. A negative predictive value (NPV) of 0.9 for predicting PD resulted in a positive predictive value (PPV) of 0.69 for predicting CR or PR. The prediction model was not able to separate the patients with SD from those with PD (AUC without miRs = 0.69, with miRs = 0.72, p = 0.37, Fig 2B) The re-estimated prediction model for separating patients with treatment response from those with SD included miR-17-5p, miR-92a-3p, miR-92b-3p and miR-98-5p. Adding those miRs to clinicopathological factors increased the AUC for predicting treatment response significantly from 0.79 to 0.90 (p = 0.02) (Fig 2C). A NPV of 0.9 for predicting SD, resulted in a PPV of 0.78 for predicting treatment response.

**Fig 3:**
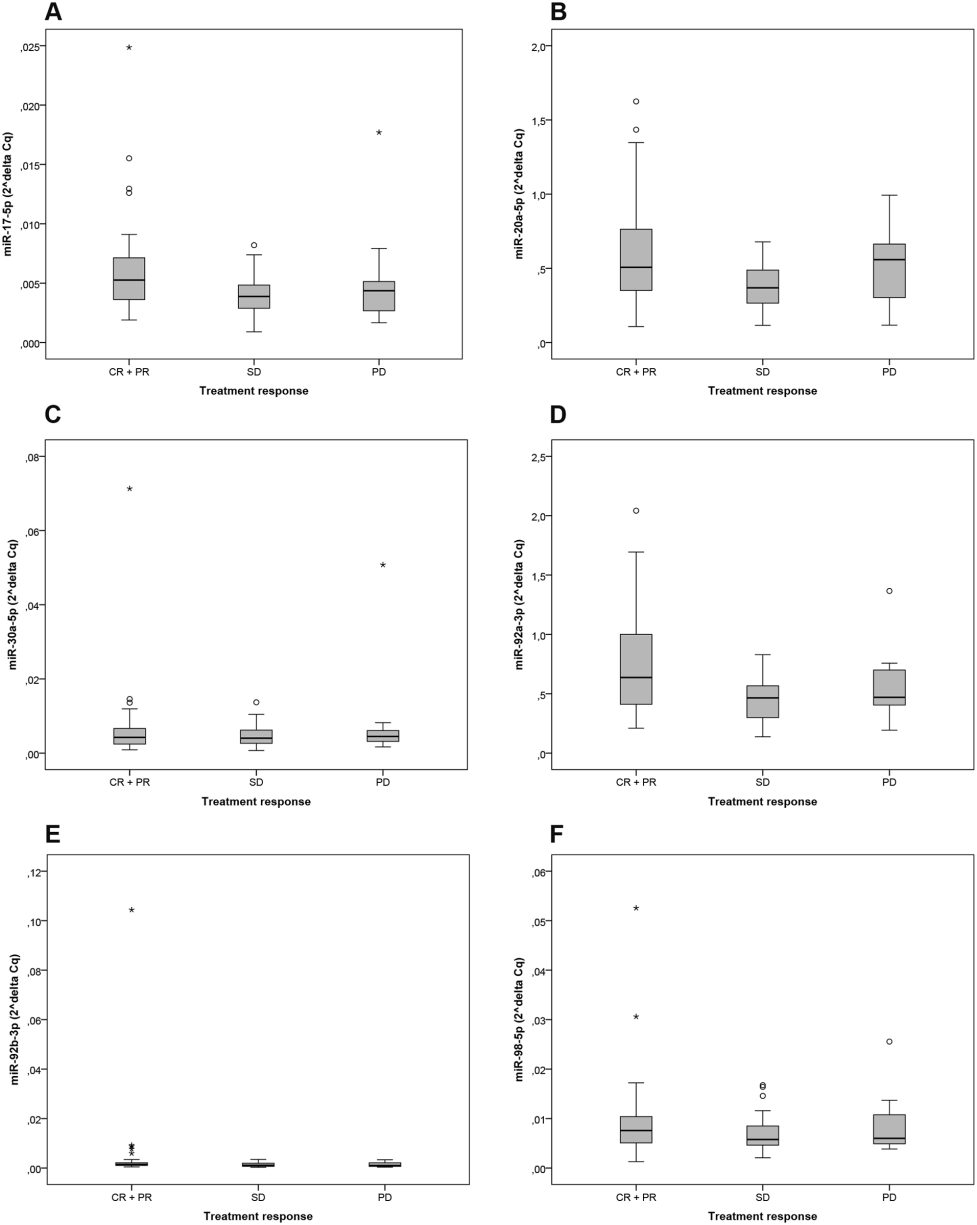
Box-plots of the expression levels of selected miRs in the validation cohort. Expression levels of **(A)** miR-17-5p, **(B)** miR-20a-5p, **(C)** miR-30a-5p, **(D)** miR-92a-3p, **(E)** miR-92b-3p and **(F)** miR-98-5p for patients with PR or CR, those with SD and those with PD. Median delta Cq values were normalized to miR-16-5p. MiR-17-5p is significantly higher expressed in patients with response compared to patients with SD (p = 0.004), but not with PD (p = 0.108). Also miR-20a-5p and miR-92a-3p are significantly higher expressed in patients with response compared to patients with SD (p = 0.006 and p = 0.005), but not with PD (p = 0.790 and p = 0.179). MiR-30a-5p, miR-92b-3p and miR-98-5p were not significantly differently expressed between the three groups.

Normalized expression levels of the six miRs tested in the validation cohort are shown in Fig 3. MiR-17-5p was significantly higher expressed in patients with treatment response compared to patients with SD (p = 0.004), but not with PD (p = 0.108). Also miR-20a-5p and miR-92a-3p were significantly higher expressed in patients with treatment response compared to patients with SD (p = 0.006 and p = 0.005, respectively), but not with PD (p = 0.790 and p = 0.179, respectively) (Fig 3). In concordance with the training cohort, miR-30a-5p, mir-92b-3p and miR-98-5p were not significantly differentially expressed between the three groups in the validation cohort.

## Discussion

CRC is a biologically heterogeneous disease due to the accumulation of genetic and epigenetic alterations over time. It has been established that CRCs with an identical genetic make-up will behave in a similar way [36–38]. In this study, we found that tumors of patients with mCRC may be separated into biological subgroups with a different response to fluoropyrimidine containing first line systemic treatment based on miR expression levels and four clinicopathological variables. When the expression levels of six miRs (miR-17-5p, miR-20a-5p, miR-30a-5p, miR-92a-3p, miR-92b-3p and miR-98-5p) are added to the clinicopathological variables (age and primary tumor differentiation, prior use of adjuvant therapy and the type of systemic treatment regimen), the AUC for identifying patients with clinical benefit increased from 0.35 to 0.78 in the training cohort. However, we were not able to validate the added predictive value of these miRs for all three response groups (treatment response *versus* SD *versus* PD) when re-estimating their predictive value in an independent validation cohort. This may be partially explained by the difference in predictive power of the four clinicopathological factors between the training cohort and the validation cohort. Clinicopathological factors did not have predictive power in the training cohort, while in the validation cohort patient could already be classified based on clinicopathological factors alone. In the validation cohort, the AUC for separating patients with treatment response from patients with SD increased significantly when adding the expression of miR-17-5p, miR-92a-3p, miR-92b-3p and miR-98-5p to the four clinicopathological factors. MiR-20a-5p and miR-30a-5p did not add predictive value to the other four miRs. This is in line with previous findings [24, 39], as not all miRs identified in a training cohort will add predictive value when re-estimating their predictive value in an independent validation cohort. The increase in AUC for predicting treatment response *versus* PD and for predicting SD *versus* PD was not significant in the validation cohort, which may be partially explained by the relative high predictive power of the clinicopathological factors alone for separating patients with treatment response from those with PD (AUC = 0.85). Although the addition of miR expression levels resulted in an AUC of 0.90 for this comparison, this improvement was not statistically significant. Clinicopathological factors alone yielded less predictive value for separating patients with treatment response from those with SD (AUC = 0.79). However, these patients could be separated with a significantly higher predictive power when adding miR expression levels (AUC = 0.90). In the validation cohort patients with SD could not be separated from patients with PD and the addition of miR expression levels yielded no additional predictive power for this comparison (AUC = 0.69 and AUC = 0.72 respectively). The different predictive behaviour of the clinicopathological factors between the training and the validation cohort and between the different response groups of the validation cohort might possibly be explained by a significantly different age distribution, a significant difference in uptake of prior adjuvant therapy, a difference in metastatic tumor load or a different distribution of rectal and colon tumors between the two cohorts, however this has to be further explored.

In the training cohort, miR expression profiles were compared between patients with clinical benefit (defined as CR, PR or SD) and patients with PD. As SD is an intermediate phenotype between patients who respond to the treatment and patients who progress it might be more difficult to classify SD using molecular markers [33]. Therefore, in the validation cohort patients were divided into three different response groups (treatment response *versus* SD *versu**s* PD)., In this study, miR expression levels of patients with SD resembled those of patients with PD in the validation cohort, which was in contrast with the training cohort where patients with SD were separated from those with PD. This indeed indicates the difficulty of classifying an intermediate phenotype based on molecular markers. The discrepancy might be explained by several factors. Firstly, the distribution of rectal, left sided and right sided CRCs differed between the training and the validation cohort, with more rectal tumors in the validation cohort and it is well known that the genetic make-up of rectal tumors differs from right-sided and left-sided CRCs [36,40]. Secondly, the metastatic tumor load of the patients in the training cohort was less than in the validation cohort with more often liver metastases only (45.5% *versus* 29.6% respectively). Thirdly, patients in the training cohort more often received prior adjuvant chemotherapy than patients in the validation cohort (22.7% *versus* 4.9% respectively), which might have induced alterations in miR expression. Fourthly, to resemble clinical practice during the validation process, no minimal tumor cell percentage was required for inclusion in the validation cohort. This could have led to a relatively higher abundance of miRs expressed in stromal tissue in the validation cohort, contributing to a different genetic make-up of both SD groups. The difference in miR expression levels of patients with SD between the validation and the training cohorts could not be explained by a different prognosis since PFS of patients with SD was similar (6.6 months *versus* 7.1 months respectively). Also, it is unlikely that intra-tumor heterogeneity of miR expression and sampling bias played a role, as the miRs selected in this study were not significantly differentially expressed between multiple tumor locations within the same patient [17].

Up-regulated as well as down-regulated miRs play a role in the carcinogenesis of CRC [41–43]. Up-regulation of mature miRs may occur due to transcriptional activation or amplification of miR encoding genes, whereas down-regulation may result from deletion of a particular chromosomal region, epigenetic silencing, or defects in miR biogenesis. Previous studies relating miR expression to treatment response in mCRC used PCR or micro-array based platforms to identify predictive miRs in their training cohorts [18, 19, 21, 22]. Consequently, these studies were limited to the analysis of a maximum of 1367 miRs. Previous studies analysing the miR transcriptome by NGS did not correlate the obtained miR expression profiles to treatment response in mCRC [44–46]. In the current study, 2567 miRs were analysed using NGS in an unbiased manner and correlated with treatment response. The selection of miRs in the training cohort was based on their predictive performance as well as on their relative abundance in CRC tissue, which may enhance future biomarker development as miRs with a relative robust expression level will be more easy to quantify using RT-qPCR based platforms. Development of miR based biomarkers to predict treatment response in the palliative as well as in the neoadjuvant setting is of clinical relevance, as such biomarkers are currently largely lacking [9, 47]. The prediction of non-response (PD or SD) is especially important in the neoadjuvant setting as prediction of non-response will prevent treatment of patients in which systemic therapy does not result in increased resection rates for advanced disease. In this study a NPV of 0.9 for predicting non-response could be reached at a PPV of 0.69–0.78 for predicting treatment response. Therefore, the miRs identified in this study might serve as potential candidate biomarkers for predicting response to neoadjuvant treatment, which has to be further explored in studies evaluating the clinical relevance of miR based biomarkers. The potential of miR based biomarkers was demonstrated in the validation cohort using RT-qPCR on FFPE tissue without the need for a minimal tumor cell percentage. FFPE tissue specimens are readily available in clinical practice and miR expression levels are highly stable detectable in these FFPE tissue specimens [14]. In this study, patients were treated with different fluoropyrimidine containing treatment regimens. Therefore, miRs that predict response to an individual drug might have been missed [48, 49]. However, by excluding patients who underwent radiotherapy or systemic treatment less than six months before tissue sampling, effects of these treatments on miR expression profiles were minimized [50,51].

Previously, we demonstrated tumor specificity of the selected miRs for mCRC tissue compared to non-tumorous colorectal tissue, with miR-17-5p, miR-20a-5p, miR-92a-3p, miR-92b-3p and miR-98-5p being significantly upregulated and miR-30a-5p being significantly downregulated in mCRC tissue compared to non-tumorous tissue [17]. The results of this study indicated that miR-17-5p, miR-20a-5p and miR-92a-3p were also significantly upregulated in patients with response on first line treatment. Interestingly, those three miRs belong to the miR-17-92 cluster, which contains 6 oncogenic miRs collectively named as “OncomiR-1” [52]. Upregulation of this cluster was associated with adenoma to carcinoma progression [53]. Recently, a higher expression of this cluster was observed in chemosensitive compared to chemoresistant pancreatic cancer stem cells [54]. Pancreatic stem cells lost their stem-like features when the miR-17-92 cluster was overexpressed resulting in reduced self-renewal capacity and increased proliferation rate as well as chemosensitivity [54]. Our finding that overexpression of the miR-17-92 cluster may also result in chemosensitivity of mCRC is in concordance with previous reports on localized CRC, which indicated that a higher expression of miR-20a-5p was associated with a favorable response to adjuvant fluorouracil based chemotherapy [24]. However, results are not unambiguously since elevated expression of miR-17 was previously also associated with resistance to 5-FU, oxaliplatin and irinotecan by repressing PTEN expression [55].

In conclusion, this study analysed the miR transcriptome using an unbiased whole genome approach and identified a six miR expression signature with potential to improve the prediction of treatment response to fluoropyrimidine containing first line systemic treatment regimens in patients with mCRC. The identified miRs have potential to serve as candidate biomarkers for predicting treatment response, when this signature is combined with four clinicopathological factors., however their clinical relevance has to be further explored.

## Abbreviations

AIC: Akaike’s information criterion
AUC: area under the curve
CR: Complete remission
EBI: European Bioinformatics Institute
FDR: False discovery rate
FFPE: formalin fixed and paraffin embedded
mCRC: metastatic colorectal cancer
miR: microRNA
NGS: next generation sequencing
NPV: negative predictive value
OS: overall survival
PD: progressive disease
PFS: progression free survival
PPV: positive predictive value
PR: Partial remission
RT-qPCR: Reverse transcription quantitative polymerase chain reaction
SD: Stable disease
